# Occurrence and predictors of lifetime suicidality and suicidal ideation in autistic adults

**DOI:** 10.1177/13623613231225901

**Published:** 2024-02-10

**Authors:** J van Bentum, M Sijbrandij, M Huibers, S Begeer

**Affiliations:** 1 Vrije Universiteit Amsterdam, The Netherlands; 2 Utrecht University, The Netherlands

**Keywords:** autism, autism spectrum diagnoses, cohort study, occurrence, suicidal ideation, suicide

## Abstract

In the past 40 years, accumulating evidence suggested that autistic individuals are at an increased risk of suicidal thoughts and behaviors. This study examined the occurrence of various potential risk factors for lifetime suicidal behavior and suicidal thoughts in the past month in a Dutch cohort (Netherlands Autism Register) of autistic individuals using the Suicidal Behaviors Questionnaire (SBQ-R) and the Suicidal Ideation Attributes Scale (SIDAS). Our results show that the majority (80%) of the initial sample (*N* = 1164) had thought about or attempted suicide in their lifetime, of which 15% had attempted suicide. In the final sample (*n* = 421), backward linear regression analyses identified the following predictors for suicidal behavior: psychiatric comorbidity, loneliness, and higher number of autistic traits, *F*(3, 240) = 21.22, *p* < 0.001, with *R*^2^ of 0.21. For suicidal thoughts in the past month, psychiatric comorbidity and a higher number of autistic traits were significant predictors, *F*(2, 241) = 20.34, *p* < 0.001, with *R*^2^ of 0.14. In sum, additional risk markers for suicidal thoughts and behaviors in autistic individuals (e.g. number of autistic traits) should be considered when assessing the suicide risk in autistic individuals. Future research should focus on adapting suicide prevention interventions for autistic individuals, such as addressing loneliness as one of the intervention strategies.

**Lay Abstract:**

Over the past few years, more and more research is showing that many autistic people are at an increased risk for suicide. In this study, we asked participants from the Netherlands Autism Register, which is longitudinal register including individuals with autism, about their possible experiences with thoughts and feelings about suicide. Specifically, we looked at whether these thoughts and feelings in their lifetime and in the past month were related to various factors (such as their age, gender, and having psychiatric disorder diagnoses). We found that 80% of the participants had experienced thoughts about or even attempted to take their own life at least once throughout their lifetime. Furthermore, in a subgroup of participants, we found that the presence of a psychiatric disorder diagnosis, feelings of loneliness, and a higher number of autistic traits were associated with experiencing suicidal thoughts and feelings in their lifetime. For those who experienced these suicidal thoughts in the past month, we found that having (multiple) psychiatric disorder diagnoses and a higher number of autistic traits were related to more severe and frequent thoughts about suicide in the past month. Our findings show that additional factors in autistic individuals should be considered when assessing the suicide risk, and it brings us one step closer to understanding why suicide is more common for autistic people.

Accumulating evidence suggests that people with autism spectrum diagnoses (referred to collectively here as autism)^1^ are at an increased risk of thinking about and attempting suicide (Hedley et al., 2018; Howe et al., 2020; Kirby et al., 2019; Newell et al., 2023; Segers & Rawana, 2014; Zahid & Upthegrove, 2017). Suicide can be defined as the act of deliberately killing oneself, and the term suicidal behavior encompasses thinking about suicide (ideation), planning for suicide, and non-fatal and fatal suicide attempts (World Health Organization, 2021). In a recent meta-analysis, autistic and possibly autistic individuals showed a significantly elevated pooled prevalence for suicidal ideation (34.2%; 95% CI 27.9–40.5), suicide plans (21.9%; 95% CI 13.4–30.4), and suicidal attempts and behaviors (24.3%; 95% CI 18.9–29.6; Newell et al., 2023). In comparison, national surveys reveal that 8.3% of the Dutch general population has had suicidal thoughts at some point in their lives, 3.0% have made a suicide plan, and 2% to 3% have made a suicide attempt at least once in their lives (ten Have et al., 2011). Research has reported that there may be differences in how suicidal thoughts and behaviors develop and that risk markers may operate differently for autistic and non-autistic individuals (Cassidy et al., 2018; Pelton et al., 2020). Therefore, this study aims to explore the incidence rates of lifetime suicidal behavior and suicidal thoughts in the past month in Dutch autistic adults, as well as particular risk factors associated with suicidal behavior and thoughts in autism.

Thus far, risk factors for suicide in the general population have been well established (Franklin et al., 2017; Nock et al., 2016). These include prior suicide attempts, exposure to suicide, misuse and abuse of alcohol, access to lethal means, occurrence of mental health conditions (especially depression and other mood disorders), and social isolation (World Health Organization, 2021). The risk factors can vary by age group, culture, sex, and other characteristics such as belonging to a group that is vulnerable to experiencing discrimination (e.g. refugees, sexual minorities, or prisoners) (Canetto & Sakinofsky, 1998; Hunt et al., 2006).

Particularly the co-existence of multiple psychiatric disorders (also referred to as “psychiatric comorbidity”) is a strong predictor of suicidal behavior in the general population (Auerbach et al., 2019; Henriksson et al., 1993; Nock et al., 2010). In recent years, various studies have revealed that the presence of multiple psychiatric disorders also heightens suicide risk in autistic people (Hand et al., 2020; Hwang et al., 2019; Moseley et al., 2022; O’Halloran et al., 2022). One study showed that 72.5% of their sample with autism had received a diagnosis of psychiatric disorders (Kõlves et al., 2021). At the same time, another study revealed that the increased risk for intentional self-harm among autistic subjects decreased to non-significant levels after adjusting for comorbid psychiatric disorders (Jokiranta-Olkoniemi et al., 2021). In terms of mental health at symptom-level, the latest network analyses uncovered that individuals with autism reported more frequent suicidal thoughts, anxiety, and depression, than non-autistic adults (Pelton et al., 2023). We want to explore this potential risk factor further in the current Dutch cohort.

Loneliness is another risk factor in the general population that is consistently associated with suicidal ideation and behavior (McClelland et al., 2020; Shaw et al., 2021; Stickley & Koyanagi, 2016). A common misconception is that autistic individuals prefer social isolation (Mazurek, 2014). On the contrary, they often want to develop relationships but may experience social difficulties resulting in unfulfilled social needs (Bauminger et al., 2003; Ee et al., 2019; Hedley et al., 2018). This has been a topic of interest in recent studies (Hedley et al., 2018; Schiltz et al., 2021), and a recent systematic review reported various factors associated with increased loneliness in autistic adults such as autistic characteristics, negative experiences and learned helplessness, anxiety, and depression and suicidal ideation (Grace et al., 2022). We also expect to find an association between loneliness and increased suicidal behavior in our Dutch cohort. A recent study in this cohort revealed that autistic adults’ loneliness and stress levels remained stable over time but were consistently higher than those of non-autistic adults (Scheeren et al., 2022).

There seems to be an association between suicide and self-rated autistic traits (McDonnell et al., 2020; Veenstra-VanderWeele, 2018). Autism-related variables include feelings of less/fewer interpersonal skills, social camouflaging (i.e. attempts to actively mask and compensate for autistic traits in social situations to ‘fit in’; Cassidy, Gould, et al., 2020), and impulsivity (Moseley et al., 2020; Pelton & Cassidy, 2017; Pelton et al., 2020). A recent study in a non-clinical, general population revealed that people with high autistic traits were more likely to experience feelings that they do not belong in this world or are a burden on others, which may increase their likelihood of attempting suicide (Pelton & Cassidy, 2017). Autistic persons may also experience rigidity and difficulties generating alternative solutions to problems in their daily lives (Paquette-Smith et al., 2014; South et al., 2020), leading to feelings of being trapped (Cassidy, Robertson, et al., 2020) and ultimately seeing suicide as the only possible escape (Baumeister, 1990; Ringel, 1976). Core aspects of an autism diagnosis may provide a risk for suicide—independent of comorbid conditions; however, this has not been reflected in the existing literature (Hedley et al., 2021).

Unemployment is common among autistic individuals, despite having the ability and desire to work (Ohl et al., 2017). While it is theorized that unemployment may be related to suicide (Hedley et al., 2017; Pelton & Cassidy, 2017), one study found that approximately half (49%) of the autistic individuals who completed suicide were listed as having a job or being a student (Kirby et al., 2019). This might suggest that autistic individuals who are employed are not necessarily at lower risk for suicide (Kirby et al., 2019). While the rate of suicide attempts was highest for employed individuals: 3.89-fold higher for those with autism compared to those without (Kõlves et al., 2021), the unemployed autistic individuals still had a 2.24-fold higher incidence rate of suicide attempts compared with those who were employed (Kõlves et al., 2021).

Moreover, Camm-Crossbie et al. (2019) report that autistic people are not believed when help-seeking as they may be managing to retain a routine, such as employment. Nevertheless, being employed does not equate that the individual is coping or no longer needs support or treatment (Camm-Crosbie et al., 2019). Moreover, autistic people are often unhappy in their working environments, which may contribute to stress and mental illness (Romualdez et al., 2021). Thus, more research is needed to explore the impact of employment in relation to suicide risk in an autistic population.

Finally, demographic characteristics such as age (Shah, 2007) and sex (at birth) seem to be significant predictors in the general population (Hunt et al., 2006). This might be similar in autistic individuals as Kirby et al. (2019) report a significant effect of age, and previous research has shown that autistic females are more likely to die by suicide than non-autistic females (Cassidy et al., 2014; Hirvikoski et al., 2016). However, there seems to be little agreement on sex differences in suicidal thoughts and behaviors as some studies find higher rates of death by suicide in females (Hirvikoski et al., 2020; Kirby et al., 2019; Kõlves et al., 2021), while others find similar levels of suicidal thoughts and behaviors (Cassidy et al., 2018; Pelton et al., 2020).

All in all, the aim of the current cross-sectional study as part of a longitudinal online cohort study was to (1) evaluate lifetime suicidal behavior (including lifetime suicidal ideation and attempts, recent frequency of ideation, suicide threats, and the likelihood of future suicidal behaviors) as well as suicidal ideation experienced in the past month in a large Dutch cohort study (Netherlands Autism Register; NAR), and (2) explore whether existing risk factors (demographic and clinical characteristics including age, sex, employment status, presence of psychiatric comorbidity, loneliness, and autistic traits) were associated with suicidal behavior and suicidal thoughts in this sample. In line with existing research, we hypothesized that age, female sex at birth, psychiatric comorbidity, employment, and loneliness were significant predictors of lifetime suicidal behavior.

## Method

### Study design

Data for this study were collected in the context of a longitudinal online database (the Netherlands Autism Register (NAR); https://www.nederlandsautismeregister.nl) that was started in 2013. The NAR consists of individuals with a *Diagnostic and Statistical Manual of Mental Disorders* (4th ed., text rev.; *DSM*-IV-TR) or (5th ed.; *DSM*-5) autism diagnosis in the Netherlands. In 2021, a total of 1220 adults (ages 16 years and older) were actively participating in the register. For the current study, questions were answered as part of a broader annual survey.

### Participants

The current sample was restricted to participants who were able to complete online self-report surveys. Inclusion criteria were minimum age of 16 years, an autism spectrum diagnosis, and proficiency in the Dutch language. The initial sample of this study included 1164 participants who answered the first item of the Suicidal Behavior Questionnaire (SBQ-R; Osman et al., 2001): “Have you ever thought about or attempted to kill yourself?” that was part of the standard database study (annual online survey). Next, participants were asked to provide additional online consent to answer the remaining three items of the SBQ-R (Items 2–4) and all five items of the Suicidal Ideation Attributes Scale (SIDAS; van Spijker et al., 2014), and whether they consented to use their data from the broader annual survey. This resulted in the final sample of 421 participants aged between 17 and 78 (*M*: 45.5, standard deviation (*SD*): 12.9 years). The final sample (*n* = 421) demographics can be found in Table 1.

**Table 1.** Sample demographics of the final sample (*N* = 421) and the retained sample (*n* = 244) included in the analyses.

Variable	Label	Final sample (*N* = 421)		Retained sample (*n* = 244)	
		*n*	*%*	*n*	*%*
Education	Low educational level	7	1.7	3	1.2
	Middle educational level	128	30.4	69	28.3
	High educational level	165	39.2	103	42.2
	*Missing/Other*	121	28.7	69	28.3
Gender	Male	109	25.9	66	27
	Female	162	38.5	99	40.6
	Other	58	13.8	37	15.2
	*Missing/not reported*	92	21.9	42	17.2
Psychiatric Comorbidity	Yes	255	60.6	155	63.5
	No	146	34.7	89	36.5
	*Missing/not reported*	20	4.8	0	0
Currently employed	Yes	202	48.0	112	45.9
	No	219	52.0	132	54.1
Currently in a relationship	Yes	211	50.1	114	46.7
	No	206	48.9	127	52
	*Don’t know/Unsure*	4	1.0	3	1.2
Has children	Yes	154	36.6	76	31.1
	No	256	60.8	167	68.4
	*Missing/not reported*	11	2.6	1	0.4

Low educational level has been defined as no former education or special lower education or primary school or practical training school; middle educational level has been defined as completing lower general secondary education or higher general secondary education or intermediate vocational education; higher education level has been defined as completing higher vocational education or pre-university education or university.

### Procedure

**Figure 1.**
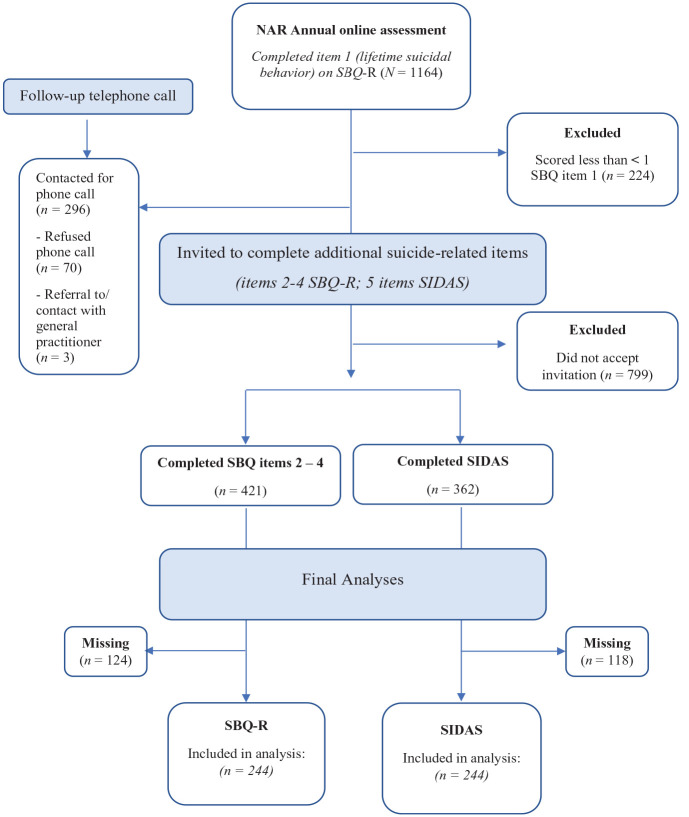
Participant flow chart. Note that there is an overlap in participants that completed the SBQ items and SIDAS items. Missing values were excluded, resulting in 244 participants included in the final analyses. NAR: Netherlands Autism Register; SBQ: Suicidal Behavior Questionnaire; SIDAS: Suicidal Ideation Attributes Scale.

Individuals could register themselves voluntarily by completing a registration form on the NAR website. Once registered, participants received a link to complete the annual online assessment (between February 2021 and April 2021), including questions regarding personal background, parent(s) and other family, problems with autism, diagnosis, and more. Furthermore, information about demographic and clinical characteristics including autism spectrum diagnosis, psychiatric comorbidities (separate item), treatment (separate item), relationships, and overall well-being was asked. This broader annual online assessment included the SBQ-R item 1 (lifetime suicidal behavior). Participants who scored > 1 on the SBQ-R item 1 were invited to the remaining items regarding their possible experiences with suicidal behavior and suicidal ideation (duration: 5 minutes). See Figure 1 for a detailed participant flow chart.

Participants who scored > 20 on the SIDAS or indicated that they wanted to receive a follow-up phone call were contacted. Prior to the phone call, an e-mail with detailed instructions regarding the purpose of the phone call, when to expect it, and contact information. The previously consulted community members recommended this procedure. During this phone call, to check the well-being of a participant, a suicide risk assessment was performed by trained research assistants; participants were provided with possible referral options and, if needed, were advised to contact their general practitioner.

The scientific and ethical review board of the Vrije Universiteit Amsterdam approved this research (VCWE-2020-162), and the study was preregistered at Open Science Framework (10.17605/OSF.IO/QF7NY). Furthermore, the investigation was carried out in accordance with the latest version of the Declaration of Helsinki.

### Measures

#### Demographic and clinical characteristics

Participants were asked to provide information regarding their age (in years), sex (at birth), presence of multiple psychiatric diagnoses (i.e. psychiatric comorbidity; yes/no), education level, relationship status, children (yes/no), and employment status.

#### Suicidal behavior

The primary outcome of this study, the Suicidal Behaviors Questionnaire (SBQ-R; Osman et al., 2001), is a self-report questionnaire with four items that assesses the following dimensions of suicidality: lifetime suicide ideation and/or suicide attempts, frequency of suicidal ideation over the past 12 months, the threat of a suicide attempt, and the likelihood of suicidal behavior in the future. Sensitivity and specificity scores were acceptable and internal consistency was strong, Cronbach’s α = .80 (Aloba et al., 2017). In this study, total scores ranged between 4 and 18, and item 1 is used as a screener’s question. If the participant scored > 1, the other three items were asked.

#### Suicidal thoughts

The secondary outcome of this study, the Suicidal Ideation Attributes Scale (SIDAS; van Spijker et al., 2014) is a self-report instrument measuring the presence and severity of suicidal thoughts. Five items assess the frequency, controllability, closeness to attempt, distress, and interference with daily activities on a 10-point scale over the past month. Total scores range between 0 and 50, with scores between 1 and 20 indicating low suicidal thoughts, and scores above 21 indicating a high risk of suicidal behavior. The SIDAS has demonstrated high internal consistency, Cronbach’s α = .91–.86 (van Spijker et al., 2014).

#### Loneliness

The Loneliness Scale (de Jong-Gierveld, & van Tilburg, 1999) is an 11-item self-report scale that evaluates the experienced loneliness. The statements are based on a loneliness model that defines loneliness as a discrepancy between what one wants in terms of interpersonal affection and intimacy, and what one has (de Jong-Gierveld, 1987). The greater the discrepancy, the greater the experienced loneliness. Participants could answer on a 5-point Likert-type scale, ranging from “1 = yes!” to “5 = no!” (e.g. “there is always someone I can talk to about my day-to-day problems”). Sum scores ranged from 11 = *not lonely* to 55 = *extremely lonely*. The scale has shown reliable and valid (Pinquart & Sorensen, 2001), and can be used as a one-dimensional measure or can be divided into two subscales (“emotional loneliness” and “social loneliness”) (de Jong-Gierveld & van Tilburg, 1999).

#### Autistic traits

The Autism Quotient Short questionnaire (AQ-Short 2011; Hoekstra et al., 2008) is a shortened version of the Autism Quotient (Hoekstra et al., 2008), a self-report questionnaire on autistic traits. The AQ-Short consists of 28 statements and participants could answer on a four-point scale, ranging from “1 = definitely disagree” to “4 = definitely agree.” Thirteen items had reverse scoring where a “disagree” response is characteristic for autism. Total AQ-Short scores were calculated by summing the items and ranged between 28 and 112. Higher scores indicated increased autistic trait severity. The items can be assigned to five defined factors: assessing difficulties with social skills, preference for routine, attention switching difficulties, difficulties with imagination, and a fascination for numbers/patterns. The AQ showed acceptable to good internal consistency: Cronbach’s α = .77–.82 (Hoekstra et al., 2011).

### Data analysis

The occurrence of lifetime behavior and suicidal thoughts (primary outcomes) was explored using descriptive statistics. Next, sociodemographic characteristics among the final sample (i.e. participants that reported suicidal thoughts in the past month, *n* *=* 421) were explored. Prior to the regression analyses, assumptions of linearity, multicollinearity, and homoscedasticity were checked. No outliers based on the Mahalanobis Distance (Mahalanobis, 1936), Cooks Distance (Cook, 1977), and Leverage Point tests were detected.

A backward linear regression was used to identify a selection possible predictors of lifetime suicidal behavior and suicidal thoughts out of the following selection of candidate variables: age, sex, employment status, loneliness, the number of autistic traits, and psychiatric comorbidity. This method was chosen to prevent coincidence findings, as this method starts with all variables corrected for each other. At each step, the least significant variable was removed until only significant variables (i.e. *p* < 0.01, to correct for multiple testing) remained. Another backward linear regression was used with the same candidate variables as the previous one, but with suicidal thoughts in the past month as the secondary outcome variable. All analyses were performed with SPSS v. 27 (SPSS, USA, Inc).

### Community involvement

For the current study, an inclusive research approach was adopted. A community member was consulted and a panel was asked about suggestions regarding the dissemination of questions concerning this particular topic. Overall, the content and formulation of the annual NAR survey are inspired by needs and interests expressed by stakeholders from the autism community, including autistic adults, parents and the Dutch Association for Autism (NVA), an autism advocacy group. Every year a panel of stakeholders (autistic adults and parents of children with varying abilities) is invited to discuss and exchange ideas on relevant research topics, methodology and dissemination of findings. The NAR also has several autistic team members (see Supplemental Appendix 1 for a detailed description of community involvement).

## Results

### The occurrence of lifetime suicidal behavior and suicidal thoughts in the past month

In total, 1164 out of 1220 individuals registered in the NAR, completed the online survey. Our results showed that 80% of the participants in our (initial) sample (*N* = 1164) had thought about or attempted suicide in their lifetime (see Table 2). Only 224 participants (19.2%) had never thought about or attempted suicide. Half (50.3%) of the final sample (*n* = 421) had suicidal thoughts in the past month (SIDAS scores).

**Table 2.** The occurrence of lifetime suicidality using the SBQ-R (*N* = 1164) and SIDAS (*n* = 421) in the Netherlands autism register.

SBQ-R item	Item choices	*n*	%
1. Lifetime suicidality			
	Never	224	19.2
	Just a brief passing thought	314	27.0
	Had a suicide plan	451	38.7
	Attempted suicide	175	15
	*Total*	*1164*	
2. Frequency of suicidal ideation in past year			
	Never	149	36.2
	Rarely (1 time)	82	19.9
	Sometimes (2 times)	78	18.9
	Often (3–4 times)	34	8.3
	Very often (5 or more times)	69	16.7
	*Total*	*412*	
3. Threat of suicide attempts			
	No	166	40.3
	Yes, at one time	113	27.5
	Yes, more than once	133	32.2
	*Total*	*412*	
4. Self-reported likelihood of suicidal behavior in the future			
	Never	55	13.3
	No chance at all	68	16.5
	Rather unlikely	164	39.8
	Unlikely	64	15.5
	Likely	39	9.5
	Rather likely	11	2.7
	Very likely	11	2.7
	*Total*	*412*	
SIDAS Sum Score	Item choices	*n*	%
Suicidal ideation in past month		*N*	%
	No ideation (0)	180	49.7
	Low ideation (1–20)	143	39.5
	High ideation (21–50)	39	10.8
	*Missing*	*59*	
	*Total*	*421*	

SBQR: Suicidal Behavior Questionnaire-Revised; SIDAS: Suicidal Ideation Attributes Scale.

Suicidal ideation in past month based on SIDAS.

### Risk factors for lifetime suicidal behavior

The backward stepwise linear regression model included the factors: age, sex, loneliness, psychiatric comorbidity (present/not present), the number of autistic traits (AQ-short), and employment status (see Table 3 for a summary of all models). Note that missing (at random) values were excluded, resulting in 244 participants included in the analysis. Model 1, including all possible predictor variables, was significant, *F*(6, 237) = 11.88, *p* < 0.001, with an *R*^2^ of 0.23. Based on the selection rule (remove variable when *p* > 0.01), age, sex, and employment status were removed. The fourth and final model, was significant, *F*(3, 240) = 21.22, *p* < 0.001, with an *R*^2^ of 0.21. Thus, the final model included: number of autistic traits, loneliness, and psychiatric comorbidity as statistically significant predictors of lifetime suicidal behavior.

**Table 3.** Summary of backward stepwise linear regression analysis for risk factors predicting lifetime suicidal behavior (SBQ-R) (*n* = 244).

Models	Predictor variables	*B*	*SE B*	*t*	*p*	95% CI B		*R* ^2^	Adjusted *R*^2^	*p*
						Lower	Upper			
Model 1	Age	−0.02	0.02	−1.08	0.280	−0.05	0.01	0.231	0.212	<0.001
	AQ scores	0.06	0.02	3.41	<0.001**	0.03	0.10			
	Empl. status	0.47	0.39	1.21	0.226	−0.30	1.24			
	Loneliness	0.08	0.02	4.03	<0.001**	0.04	0.12			
	Sex	0.65	0.42	1.56	0.121	−0.17	1.47			
	Psyc. comorbidity (yes)	−1.75	−0.26	−4.24	<0.001**	−2.57	−0.94			
Model 2	AQ scores	0.06	0.02	3.26	0.001**	0.02	0.09	0.227	0.211	<0.001
	Empl. status	0.43	0.39	1.10	0.274	−0.34	1.19			
	Loneliness	0.08	0.02	3.95	<0.001**	0.04	0.12			
	Sex	0.79	0.39	2.00	0.046*	0.01	1.57			
	Psyc. comorbidity (yes)	−1.79	0.41	−4.34	<0.001**	−2.60	−0.98			
Model 3	AQ score	0.06	0.02	3.31	0.001**	0.02	0.09	0.223	0.210	<0.001
	Loneliness	0.08	0.02	3.98	<0.001**	0.04	0.12			
	Sex	0.81	0.39	2.06	0.040*	0.04	1.59			
	Psyc. comorbidity (yes)	−1.91	0.40	−4.80	<0.001**	−2.69	−1.13			
Model 4	AQ score	0.06	0.02	3.32	0.001**	0.02	0.09	0.210	0.200	<0.001
	Loneliness	0.08	0.02	3.75	<0.001**	0.04	0.12			
	Psyc. comorbidity (yes)	−2.11	0.39	−5.43	<0.001**	−2.87	−1.34			

SE: standard error; CI: confidence interval; Psyc. comorbidity: psychiatric comorbidity; Empl. Status: employment status.

Employment status was coded as: 0 = unemployed, 1 = employed. Sex was coded as: 0 = male, female. Psychiatric comorbidity was coded as 0 = no, 1 = yes. At each step, the least significant variable was removed until only significant variables (i.e. *p* < .01, to correct for multiple testing) remained. Negative values (such as for Psyc. comorbidity) indicate an inverse relationship.

* *p* < .05; **p < .01.

### Risk factors for suicidal thoughts in the past month

Another backward stepwise linear regression model was used to identify whether the same set of variables were predictors of suicidal thoughts in the past month in autistic individuals. A summary of the five regression analyses is provided in Table 4. Note that missing (at random) values were excluded, resulting in 244 participants included in the analysis. Model 1, including all possible predictor variables was significant, *F*(6, 237) = 8.77, *p* < .001), with an *R*^2^ of .182. For the fifth and final model a significant regression equation was found, *F*(2, 241) = 20.34, *p* < .001, with an *R*^2^ of .144, leaving number of autistic traits and psychiatric comorbidity as significant predictors of suicidal thoughts in the past month.

**Table 4.** Summary of backward stepwise linear regression analysis for risk factors predicting suicidal thoughts in the last month (SIDAS) (*n* = 244).

Models	Predictor variables	*B*	*SE B*	*t*	*p*	95% CI B		*R* ^2^	Adjusted *R*^2^	*p*
						Lower	Upper			
Model 1	Age	−0.07	0.05	−1.48	0.142	−0.16	0.02	0.182	0.161	<0.001
	AQ score	0.18	0.05	3.29	0.001**	0.07	0.28			
	Empl. status	2.00	1.19	1.68	0.095	−0.35	4.34			
	Loneliness	0.16	0.06	2.58	0.010*	0.04	0.29			
	Sex	−0.44	1.27	−0.35	0.728	−2.94	2.06			
	Psyc. comorbidity (no)	−5.21	1.26	−4.13	<0.001**	−7.70	−2.73			
Model 2	Age	−0.07	0.05	−1.44	0.151	−0.15	0.02	0.181	0.164	<0.001
	AQ score	0.18	0.05	3.28	0.001**	0.07	0.28			
	Empl. status	1.96	1.18	1.66	0.099	−0.37	4.29			
	Loneliness	0.16	0.06	2.62	0.009**	0.04	0.29			
	Psyc. comorbidity (no)	−5.13	1.24	−4.14	<0.001**	−7.57	−2.69			
Model 3	AQ score	0.16	0.05	3.10	0.003*	0.06	0.27	0.174	0.160	<0.001
	Empl. status	1.81	1.18	1.53	0.127	−0.52	4.13			
	Loneliness	0.15	0.06	2.46	0.015*	0.03	0.28			
	Psyc. comorbidity (no)	−5.40	1.23	−4.40	<0.001**	−7.81	−2.98			
Model 4	AQ score	0.17	0.05	3.12	0.002**	0.06	0.27	0.166	0.156	<0.001
	Loneliness	0.16	0.06	2.49	0.013*	0.03	0.28			
	Psyc. comorbidity (no)	−5.93	1.18	−5.03	<0.001**	−8.25	−3.61			
Model 5	AQ score	0.17	0.05	3.25	0.001**	0.07	0.28	0.144	0.137	<0.001
	Psyc. comorbidity (no)	−6.16	1.19	−5.19	<0.001**	−8.50	−3.82			

SE: standard error; CI: confidence interval; AQ: autism quotient; Psyc. comorbidity = psychiatric comorbidity; Empl. Status = employment status.

Employment status was coded as: 0 = unemployed, 1 = employed. Sex was coded as: 0 = male, female. Psychiatric comorbidity was coded as 0 = yes, 1 = no. At each step, the least significant variable was removed until only significant variables (i.e. *p* < .01, to correct for multiple testing) remained. Negative values (such as for Psyc. comorbidity) indicate an inverse relationship.

* *p* < .05; **p < .01.

## Discussion

This study examined the occurrence of lifetime suicidal behavior and suicidal thoughts in the past month in a Dutch cohort of autistic individuals and explored various potential risk factors for suicide. Our results showed that the majority (80%) of the full sample (*N* = 1164) had thought about or attempted suicide in their lifetime, of which 38.7% had made a suicide plan and 15% had attempted suicide. Moreover, half of the sample (50.3%) responding to our additional questionnaires (*n* = 421) had experienced suicidal thoughts in the past month. For lifetime suicidal behavior, we found the following predictor variables: psychiatric comorbidity, loneliness, and the number of autistic traits. For suicidal thoughts in the past month, psychiatric comorbidity and the severity of autistic traits were significant predictor.

In the initial sample, the lifetime experience of suicidal behavior and plans was 10-fold higher than in the general Dutch population (ten Have et al., 2011). Our results also show higher rates of suicidal behavior among autistic individuals compared to other studies among autistic individuals, showing rates of suicide attempts and suicidal thoughts varying from 3.8% to 66% (Zahid & Upthegrove, 2017). It should be pointed out that our study sample may not be representative for the Dutch autism population as the cohort included relatively more highly educated individuals and more females, reducing the generalizability of the findings. Furthermore, questions that explicitly asked about suicide were optional (as part of the broader annual survey), which could lead to an overrepresentation of participants willing to enclose information about their suicidal experiences. Actual numbers might be somewhat milder in the Dutch autism population.

Psychiatric comorbidity increased suicide risk (Auerbach et al., 2019; Henriksson et al., 1993; Nock et al., 2010). One explanation for this finding may be that the presence of multiple psychiatric disorders, on top of the impaired functioning due autism-related traits, may increase levels of distress and experienced burden (Angst et al., 2002; Nock et al., 2010). Thus, our study adds to these results reporting that psychiatric comorbidity may be an important risk factor for suicide in autistic individuals, potentially due to the accumulation and interplay of various psychiatric symptoms present.

Loneliness predicted lifetime suicidal behavior scores but not suicidal thoughts in the past month. This is consistent with findings that loneliness was more strongly associated with suicidal behavior in the longer term than in the short term (McClelland et al., 2020). Autism-related traits may enhance the potential for feeling lonely if mutual difficulties in social communication and understanding, and social interactions between autistic people and neurotypical people are experienced repeatedly over a long period (Davis & Crompton, 2021; Ee et al., 2019; Hedley et al., 2018). This is in line with the double empathy problem (Milton & Sims, 2016; Mitchell et al., 2021), which proposes that rather than a deficiency of autistic individuals in socializing, there are bidirectional differences in communication style and social-cognitive characteristics between the two groups. Furthermore, according to the Interpersonal Psychological Theory of Suicide (Joiner, 2005) loneliness and the absence of emotional support can lead to self-destructive behaviors (Shaw et al., 2021; van Orden et al., 2010). Individuals with autism, particularly, are more likely to report situations associated with thwarted belonging, such as social isolation and loneliness (Haertl et al., 2013; Mazurek, 2014).

In addition, our data collection occurred during the COVID-19 pandemic, which may have affected these loneliness levels. However, a recent study using the same NAR database indicated that autistic adults’ loneliness and stress levels remained stable at a consistently higher level than those of non-autistic adults. There were large person-to-person differences (Scheeren et al., 2022).

There may be an array of factors related to autism that amplify other risk factors for suicide such as ruminating thought patterns, feeling trapped when unable to generate alternative solutions to problems in their daily lives (Autistica, 2020; Paquette-Smith et al., 2014). Moreover, autistic adults experienced barriers to feeling that they belonged in various social spaces which in turn had an effect on their wellbeing (Milton & Sims, 2016). If mutual difficulties in social communication and understanding occur over a long period of time, this may increase feelings of loneliness as well period (Davis & Crompton, 2021; Ee et al., 2019; Hedley et al., 2018). Alternatively, our sample sizes for lifetime suicidal behavior and suicidal thoughts in the past month differed and may have influenced our results.

In this study, employment status was not a significant predictor of suicidal behavior. While previous research has shown that unemployment may exclude autistic individuals from acquiring and applying life and social skills, in turn having negative personal, familial, and financial effects (Solomon, 2020), other studies (Camm-Crosbie et al., 2019) have suggested that employment might bring additional stressors as autistic individuals experience more workplace bullying and encounter more stressful situations at work compared to people without autism. Employment status was relatively evenly divided between employed and unemployed in our sample. Potentially risk factors associated with unemployment (such as indirect negative personal and financial effects; Hedley et al., 2017), and risk factors associated with employment (such as additional stressors; Camm-Crosbie et al., 2019) may have played a role in our sample. A better understanding of an individual’s perceived quality of life as a result of employment needs to be considered to evaluate the effects of employment status (Scott et al., 2019; Solomon, 2020).

Contrary to our hypotheses, we did not find female sex at birth to be a significant predictor of suicidal behavior. Moreover, in our sample, there were no significant differences between autistic females and males in terms of suicidal behavior. While suicide attempt rates of males are elevated in the general population (Canetto & Sakinofsky, 1998), these rates seem to be more equally distributed between males and females with autism (South et al., 2020). Our non-significant findings align with previous studies that find similar patterns of suicidal thoughts and behaviors (Cassidy et al., 2018; Pelton et al., 2020). Thus, our findings again suggest that suicidal behavior and suicidal thoughts may not reflect similar gender-based patterns as in the general population (Pelton et al., 2020). A potential explanation for the smaller disparity in suicide rates between autistic males and females compared to the general population might be that autistic females are at an increased risk for suicide due to the different ways autism manifests in and is experienced by females (Pelton et al., 2020; South et al., 2020). Autistic females were reported to be more socially motivated but struggled to find genuine social reciprocity (South et al., 2020). Another explanation could be that females may be more inclined to mask their autistic traits to “fit in” (i.e. “social camouflaging”) (Cassidy et al., 2019). However, another study in adolescents admitted to emergency departments revealed that males were overrepresented among adolescent suicide attempters with autism (Mikami et al., 2020).

### Limitations

Some limitations of the current study must be acknowledged. First, the study had a cross-sectional design and while this may provide information about correlations, more longitudinal studies are required to identify risk factors (Franklin et al., 2017). Second, the sample was not a representative sample. For example, due to the self-report nature of the data people with limited literacy competency may have been underrepresented. The Netherlands Autism Register cohort consists of mainly higher educated individuals. Third, many participants filled out item 1 of the Suicidal Behaviors Questionnaire (SBQ-R) in the larger sample but did not complete the additional items nor the Suicidal Ideation Attributes Scale (SIDAS). Fourth, only some participants agreed to use their data from the annual online survey, which unfortunately led to quite some missing data. Moreover, for about an additional 50% of those completing the remaining, SBQ-R items and SIDAS items were not available for the prediction analyses because of missing values. Thus, while there was a large sample participating in the online database, only a quarter of the sample provided complete data. Due to this, selection bias might have occurred. One potential explanation may be that these individuals prefer not to talk about this topic, or it may be that these individuals explicitly do not want to disclose their suicidal thoughts. Finally, the SBQ-R has not been validated for autistic adults (Cassidy, Bradley, et al., 2020; Pelton et al., 2020). This is important, since measures of psychological constructs in relation to suicidal thoughts and behaviors should ideally be validated among autistic individuals to enable accurate inference about suicide risk markers or autistic individuals (Cassidy, Robertson, et al., 2020; Pelton et al., 2020). Since conducting our study, a modified version of the SIDAS has been evaluated in an autistic sample (SIDAS-M; Hedley et al., 2023).

## Conclusion and future suggestions

Our results add to the growing body of evidence regarding the increased risk of suicide in autistic people. We showed that while there seems to be some overlap with risk factors for suicide in the general population (such as the presence of psychiatric comorbidity and loneliness), it is evident that there are additional risk markers for suicidal thoughts and behaviors especially in autistic individuals. Severity of autistic traits should be considered when assessing the suicide risk in autistic persons.

Current suicide prevention screening and treatment strategies have focused mostly on targeting risk factors known to be present in the general population. However, these do not always match the needs of autistic individuals. Future research should focus on adapting suicide prevention interventions for autistic individuals for them to better address the specific challenges these individuals face. One suggestion might be to target feelings of thwarted belongingness, as it is hypothesized to be dynamic and amenable to therapeutic change (van Orden et al., 2010). Loneliness should be one of the main factors in treatment to reduce suicidal risk. Furthermore, an autistic individual’s perceived quality of life as a result of employment should be evaluated and changes in the currently often “unwelcoming” hiring environments toward autistic individuals, characterized by biases and stigma instead of a focus on the financial and social benefits of hiring autistic adults (Scott et al., 2019).

## Supplemental Material

sj-docx-1-aut-10.1177_13623613231225901 – Supplemental material for Occurrence and predictors of lifetime suicidality and suicidal ideation in autistic adults Supplemental material, sj-docx-1-aut-10.1177_13623613231225901 for Occurrence and predictors of lifetime suicidality and suicidal ideation in autistic adults by J van Bentum, M Sijbrandij, M Huibers and S Begeer in Autism

## Notes

1. The preference for “person with autism” (PFL, person-first language) and “autistic person” (IFL, identity-first language) differs (e.g. Buijsman et al., 2023). Therefore, we chose to use both interchangeably.
