# Supplementary material for: Occurrence and predictors of lifetime suicidality and suicidal ideation in autistic adults
Source: Autism. 2024 Feb 10;28(9):2282–94. doi: 10.1177/13623613231225901 (PMC11408986; doi:10.1177/13623613231225901)
Supplement: sj-docx-1-aut-10.1177_13623613231225901 – Supplemental material for Occurrence and predictors of lifetime suicidality and suicidal ideation in autistic adults [file sj-docx-1-aut-10.1177_13623613231225901.docx]

**Appendix 1 – GRIPP2 Reporting Checklist Community Involvement – Short Form**

| *Section and Topic* | *Item* | *Section where reported and details* |
| --- | --- | --- |
| 1: Aim | Report the aim of PPI in the study | The study aim of involving community members is included in the Methods: “For the current study, an inclusive research approach was adopted.”  “Overall, the content and formulation of the annual NAR survey are inspired by needs and interests expressed by stakeholders from the autism community, including autistic adults, parents and the Dutch Association for Autism (NVA), an autism advocacy group.”  We believe that community member engagement in this research is essential, in order to ensure that the participant perspectives were integral to preparation, execution and translation.  More importantly, the idea for the current study came from the autism community stressing the importance of this topic and how it was understudied in this community. Initially, insufficient opportunities for aftercare were available hence the delay in conducting the study. |
| 2: Methods | Provide a clear description of the methods used for PPI in the study | Methods of how community members were involved were discussed under the section ‘Community Involvement’.  Meetings and close contact with a community member were held throughout the study. In addition, prior to conducting the research a panel was consulted about suggestions regarding the dissemination of questions related to suicidal behavior and suicidal thoughts. |
| 3: Study results | Outcomes – Report the results of PPI in the study, including both positive and negative outcomes | The research team reflected on and discussed the outcomes, benefits, and challenges of patient engagement.  During the follow-up call moments, participants (community members) provided feedback on their participation in the study. For example, the opportunity for a follow-up call was greatly appreciated.  Furthermore, they explained how the questions regarding suicidal behavior and thoughts affected them and possible suggestions for future research. |
| 4: Discussion and conclusions | Outcomes – Comment on the extent to which PPI influenced the study overall. Describe positive and negative effects | Specific instances of community members’ influence are also included in the paper Methods and Discussion, such as: “Prior to the phone call, an e-mail with detailed instructions regarding the purpose of the phone call, when to expect it, and contact information. The previously consulted community members recommended this procedure.” |
| 5: Reflections/critical perspective | Comment critically on the study, reflecting on the things that went well and those that did not, so others can learn from this experience | Information regarding things that went well and things that did not, were discussed in the Discussion section, particularly in the Limitations.  For example, a critical reflection on the use of questionnaires that were not validated in an autistic population. This may have affected the accuracy of measuring psychological construction in relation to suicidal thoughts and behaviors in autistic individuals.  Discussions about the formulation of the questions were held with panel/community members pointed towards this limitation. |
